# Supplementary material for: Sustained-input switches for transcription factors and microRNAs are central building blocks of eukaryotic gene circuits
Source: Genome Biol. 2013 Aug 23;14(8):R85. doi: 10.1186/gb-2013-14-8-r85 (PMC4054853; doi:10.1186/gb-2013-14-8-r85)
Supplement: Additional file 5 — HTML Browsable Motif Output. Zipped folder containing all WaRSwap and FANMOD motif output, viewable in a web browser. [file gb-2013-14-8-r85-S5.ZIP › HTML_browsable_motif_output/WaRSwap_all_tair9/sigs_WSWRSLDraw-2000_all.pvals.heatmaps.html/motif_id_36_001001001_tftype_all_upstream_-2000_0.html]

```
BG_MODEL = Rnew2
MOTIF_ID = 36_001001001
TF_TYPE = all
UPSTREAM = -2000_0


PVals
FN_0.2	FN_0.4	FN_0.6	FN_0.8
dg_60.genes	0.404	0.877	0.98	0.005
dg_70.genes	0.39	0.876	0.982	0.007
dg_80.genes	0.421	0.863	0.985	0.006

ZScores
FN_0.2	FN_0.4	FN_0.6	FN_0.8
dg_60.genes	0.222	-1.14	-2.026	2.474
dg_70.genes	0.25	-1.11	-2.002	2.404
dg_80.genes	0.201	-1.101	-2.027	2.417

StDevs
FN_0.2	FN_0.4	FN_0.6	FN_0.8
dg_60.genes	2282.297	1568.593	501.694	70.85
dg_70.genes	2257.412	1580.218	505.575	71.276
dg_80.genes	2244.888	1556.31	504.267	69.708
```
